# Philosophy and the clinic: Stigma, respect and shame

**DOI:** 10.1111/jep.13755

**Published:** 2022-09-02

**Authors:** Michael Loughlin, Luna Dolezal, Phil Hutchinson, Supriya Subramani, Raffaella Milani, Caroline Lafarge

**Affiliations:** ^1^ Institute for Person‐Centred Health and Social Care, School of Biomedical Sciences University of West London London UK; ^2^ Department of Sociology, Philosophy and Anthropology University of Exeter Exeter UK; ^3^ Department of Psychology, Faculty of Health, Psychology and Social Care Manchester Metropolitan University Manchester UK; ^4^ Institute of Biomedical Ethics and History of Medicine University of Zurich Zurich Switzerland; ^5^ School of Human and Social Sciences University of West London London UK

**Keywords:** bioethics, health philosophy, interaction, respect, shame, stigma

## Abstract

Since its foundation in 2010, the annual philosophy thematic edition of this journal has been a forum for authors from a wide range of disciplines and backgrounds, enabling contributors to raise questions of an urgent and fundamental nature regarding the most pressing problems facing the delivery and organization of healthcare. Authors have successfully exposed and challenged underlying assumptions that framed professional and policy discourse in diverse areas, generating productive and insightful dialogue regarding the relationship between evidence, value, clinical research and practice. These lively debates continue in this thematic edition, which includes a special section on stigma, shame and respect in healthcare. Authors address the problems with identifying and overcoming stigma in the clinic, interactional, structural and phenomenological accounts of stigma and the ‘stigma‐shame nexus’. Papers examine the lived experience of discreditation, discrimination and degradation in a range of contexts, from the labour room to mental healthcare and the treatment of ‘deviancy’ and ‘looked‐after children’. Authors raise challenging questions about the development of our uses of language in the context of care, and the relationship between stigma, disrespect and important analyses of power asymmetry and epistemic injustice. The relationship between respect, autonomy and personhood is explored with reference to contributions from an important conference series, which includes analyses of shame in the context of medically unexplained illness, humour, humiliation and obstetric violence.

## 1 PHILOSOPHY AND THE CLINIC: STIGMA, RESPECT AND SHAME

Since its foundation in 2010,^1^ the annual philosophy thematic edition of this journal has been a forum for authors from a wide range of disciplines and backgrounds, enabling contributors to raise questions of an urgent and fundamental nature regarding the most pressing problems facing the delivery and organization of healthcare.^2^ Authors have successfully exposed and challenged underlying assumptions that framed professional and policy discourse in diverse areas, generating productive and insightful dialogue regarding the relationship between evidence, value, clinical research and practice.^3^ These lively and inspiring exchanges have included important contributions to the ‘great debate’ about clinical reasoning and decision making,^4^ advancing our understanding of the professional–patient interaction and our changing conceptions regarding objectivity, diagnosis, bias, judgement and power.^5^, ^6^, ^7^

Recent editions have focussed on the ongoing discussions concerning shared decision making, person‐centred care, patient expertise and value‐based practice,^8^, ^9^ with authors building on earlier debates regarding the integration of health and social care^10^ to advance new approaches to the clinical encounter and the developing interactions between technical and humanistic features of care.^11^

These important themes are developed further in this edition, which includes a special section on stigma, shame and respect. This section includes papers aimed at identifying, understanding and addressing discrimination in clinical settings^12^, ^13^, ^14^, ^15^, ^16^, ^17^, ^18^, ^19^ and on respect and shame in healthcare and bioethics.^20^, ^21^, ^22^

The edition opens with a number of papers that address core issues in health philosophy, which have been the preoccupation of the philosophy thematic editions of this journal for over a decade. Authors raise pertinent and challenging questions concerning our understanding of the nature of health and disease,^23^, ^24^ diagnosis and the processes of clinical decision making.^25^, ^26^ These contributions are followed by several highly original articles addressing the relationship between evidence and practice in medicine and healthcare,^27^, ^28^, ^29^ demonstrating the application of specific, philosophically informed approaches to causal reasoning with reference to some very practical contemporary health controversies.^30^, ^31^ Continuing debates on pressing issues we have emphasized in more recent thematic editions, authors explain and analyse different conceptions of ‘patient‐centred care’ and ‘patient expertise’,^32^, ^33^ discussing the role of values in shaping our understanding of the mental and medical disorder,^34^, ^35^ and the relationship between epistemic risk and nonepistemic values in the end of life care.^36^ The need to understand research, its implementation and interpretation in the broader social and political context is illustrated by a fascinating article on overdiagnosis.^37^ The edition includes stimulating and provocative discussions of shared meaning making and health literacy,^38^ precision medicine and its effects on medical epistemology,^39^ and the need to rethink our conception of ‘researcher bias’ in health research.^40^

## 2 STIGMA, SHAME AND RESPECT IN HEALTHCARE

The concluding section of the edition focuses on the closely related concepts of stigma, shame and respect and how these have an impact on health and clinical practice.^12^, ^13^, ^14^, ^15^, ^16^, ^17^, ^18^, ^19^, ^20^, ^21^, ^22^

### 2.1 Stigma

Contemporary research on stigma is generally agreed to have been initiated by the publication of the sociologist Erving Goffman's now classic book *Stigma: Notes on the Management of Spoiled Identity*, which appeared in 1963.^41^ In recent decades, stigma has become a prominent topic in healthcare, largely driven by stigma's status as a significant category of phenomena, which feature prominently in the areas of mental health, sexual health and human immunodeficiency virus (HIV).

Goffman identified stigma as a feature or attribute that is operationalized in interaction to be discrediting or degrading. The reference to interaction is important: an attribute might be unremarkable and inert in one situation, a source of pride in another, while being interactionally operationalized in yet another situation in a way that stigmatizes the bearer of the attribute by degrading or discrediting them.

For example, a person might have a prominent tattoo that in some situations is simply forgotten by them and unnoticed by others, remaining a latent feature of their life and identity. In other situations, it might be commented on and serve as a source of pride, while in others, perhaps during a job interview with a particularly conservative interview panel, the tattoo becomes interactionally operationalized in such a way as to be degrading or discrediting.

While these interactional and situational features were explicit and central to Goffman's analysis of stigma, they are often overlooked in more recent work, as authors inaccurately depict Goffman as offering a general definition of stigma as an attribute^42^, ^43^, ^44^ and neglect his discussion of the operationalization of degradation or discreditation in interaction. Robin James Smith, Paul Atkinson and Rhiannon Evans^18^ in this issue address some of the problems with contemporary analyses of stigma.

Furthermore, some prominent recent work on stigma^45^, ^46^ has sought to reorient the study of the stigma away from interactional study and phenomenological analyses of the lived experience of degradation and discreditation, and instead argued for and sought to provide structural theories of stigma. The argument underpinning such proposed reorientations is that stigma is a structural phenomenon and needs to be theorized as such. This is an ongoing discussion and one that will not be pursued here. In this special issue, the editors asked for the focus of the contributions to be on the interactional dynamics of stigma. The hope was that this focus would speak directly to health professionals, so as to help them understand these dynamics in a way that might prove useful in their own practice, and in the hope that patients’ lived experiences of discreditation and degradation would take prominence over academic theories. The section includes papers designed to open up debates on understanding and addressing stigma in a range of contexts, from the labour room^17^ to mental healthcare^16^, ^19^ and the treatment of ‘deviancy’ and ‘looked‐after children’.^15^, ^18^ Authors raise challenging questions about the development of our uses of language in the context of care,^12^, ^15^, ^16^, ^17^, ^18^, ^19^ and the relationship between stigma and important analyses of power asymmetry^12^ and epistemic injustice.^15^, ^17^

### 2.2 Shame

While stigma has been extensively written on in the context of healthcare, the relationship between stigma and shame has been discussed much less. As Phil Hutchinson and Rageshri Dhairyawan noted in 2017,^47^, ^48^ while stigma is extensively discussed in the literature on HIV, relatively speaking shame rarely features as a topic for discussion. Hutchinson and Dhairyawan^48^ went on to write that studying stigma in the absence of any discussion of shame ‘is akin to proposing strategies and methods for studying the social phenomenon of “threats” while not undertaking, nor even talking about, the fear response to threats. Just as threats are of interest to us as social phenomena because of the impact they have on our lives through the responses they engender, so stigma is of interest to us because of the responses it engenders, the way it makes us feel and behave’.

In this issue, the papers by Luna Dolezal^13^ and Phil Hutchinson^14^ discuss the stigma‐shame nexus. Stigma, or enacted discreditation and degradation, can serve as objects of shame. That means that it is part of what it is to experience this shame that one feels discredited or degraded in this way. A person who experiences a degrading interaction experiences this as shameful.

Where ‘stigma’ is a term which picks out a category of social phenomena, ‘shame’ is an emotion term. While the vernacular term ‘emotion’ and individual emotion terms such as ‘shame’ are widely understood and familiar terms employed in everyday conversation, in academic discussions in the disciplines of philosophy and psychology there is much debate about the nature of the category ‘emotion’ and about specific emotions, such as shame.

Without becoming too side‐tracked by what can seem like purely academic debates, we shall note here that there is no underlying physiological process that serves to unite all emotions as members of the category ‘emotion’, nor which serves to type‐individuate the different emotions, such as shame, fear, remorse, love, guilt, and so on.^49^, ^50^, ^51^ Candidate explanations that have been proposed include ‘patterned changes in the autonomous nervous system’, ‘neurological processes’ and ‘facial expression’; all of these have failed to explain emotion.

The failure of physiological explanations leads some philosophers and psychologists to seek to explain emotions as essentially thoughts of one kind or another. Candidate explanations for the explanatory constituents of emotion on this view have included ‘evaluative beliefs’,^52^ ‘judgements’^53^, ^54^ and ‘construals’.^55^ These explanations also face significant problems and have been similarly declared to have failed.^56^, ^57^, ^58^, ^59^

Seeking to move beyond this debate, some recent accounts of emotion have emphasized the lived experience of emotions,^60^ the enactive nature of emotion^61^ or the way in which emotions are made witnessable and accountable in interaction.^59^

While it can be tempting to think of this as a *purely* academic debate, of interest only to those contributing to it in the disciplines of philosophy and psychology, it would be a mistake to do so. Our conception of shame, what we take to be the nature of shame, will inform how we understand the production of shame in the clinic and how we select the best or most appropriate methods for studying shame.

One of the things we do know is that the person experiencing shame often seeks to withdraw or retreat from the social world. This is one of the reasons that understanding shame is important for healthcare practitioners. A person who feels shame might be more inclined to withdraw from engagement in a clinical encounter, either literally by leaving the clinic or figuratively by becoming ‘closed’, less responsive and less forthcoming with answers to (clinically) important questions. For example, questions about sexual history in a sexual health consultation might be less likely to be answered openly and honestly, if at all, by someone experiencing a pronounced shame episode. Appointments might not be attended owing to shame. These would be fruitful areas for further study, but such studies must be grounded in a good understanding of shame as a phenomenon. A better understanding of the social dynamics of shame and stigma might help the clinician avoid shaming and the deleterious consequences of that.

## 3 RESPECT AND SHAME IN HEALTHCARE AND BIOETHICS

We have already noted that stigma is a category term, and in his contribution to this section of the Philosophy Thematic issue of the JECP, Hutchinson says a little more about this: stigma is a category term, like the term emotion, and it brings together experiences of degradation, discreditation and discrimination. This list is not exhaustive, and one could add to this list of members of the stigma category, (negative) bias and disrespect. This last term, ‘disrespect’, is based on the idea that there are interactions which fail to be respectful or that are experienced as disrespectful.

One helpful way to understand ‘respect’ is that it ‘necessarily has an object: respect is always directed toward, paid to, felt about, or shown for some object’.^62^ While moral philosophers have offered different accounts of respect, Robin Dillon^63^ has argued that most agree that respect operates both as an attitude and as a behaviour. Many of the debates in moral philosophy around what respect for persons amounts to have been based on an analysis of what the concept of personhood entails. This is sometimes interpreted as deriving a conception of the person from an account of persons as moral agents or based on their ascribed moral status.^64^, ^65^ We can see this understanding in early bioethics debates where the concept of personhood is linked to moral status,^65^ for example, in abortion, brain death and assisted reproduction discussions.

The moral philosopher Stephen Darwall's notion of ‘recognition respect’,^66^ which has similarities to the philosopher Stanley Cavell's concept of ‘acknowledgement’,^67^ is particularly helpful when thinking about clinical interactions,^64^ and moving us away from reductive accounts of persons as defined in terms of their ascribed moral status. Darwall has a more expansive conception of the person that is the object of respect, where respect is not just about obligations and moral status but about acknowledging the humanity (in all its richness) of the other in our deliberations about and interactions with them.

Darwall and Cavell marked real progress, but more recent work in bioethics places the lived experience of the second person perspective centre stage, so it is not so much based on our conception of humanity (even if that is rich and nonreductionist), but on how a person experiences the interaction: Do they experience the interaction as respectful? Do they feel acknowledged as a person?

We might call this the phenomenological or existential turn in the respect for persons literature. This turn to basing respect for persons on the experiential aspect or the lived experience of being respected has been particularly significant in the context of thinking of respect for persons in healthcare encounters.^64^ Our final subtheme in this Philosophy Thematic introduces the notion of respect for persons to the discussion of shame.

Respect and shame are central to healthcare. As noted, in bioethics, the moral concept of ‘respect for persons’ has gained significance in recent times.^68^, ^69^, ^70^ While the dominant understanding of ‘respect’ in bioethics has focused on ‘autonomy’, this conception has been problematized by considering phenomenological accounts of how healthcare is experienced from a patient's point of view.^64^ Phenomenological accounts reveal that affective states, especially negative self‐conscious emotions, are central to understanding respect and disrespect within healthcare encounters. Recent research demonstrates that emotions such as shame can be a powerful force in clinical encounters, and also influence health and health outcomes.^71^

The “Respect and Shame in Healthcare and Bioethics” Workshop Series ran in late 2021 and included scholars from various disciplinary backgrounds, who critically engaged with conceptual and phenomenological understandings of respect, disrespect and shame, and their significance to healthcare and bioethics debates. The contributions which make up this subsection *Respect and Shame in Healthcare and Bioethics* come from contributors to this workshop series, including Katharine Cheston^21^ and Vania Smith‐Oka,^22^ and the section includes the contribution from Elizabeth Bromley.^20^

The contributions published here ground their analyses from diverse disciplinary frameworks with a focus on the experiential aspects, particularly from shaming, shame and humiliating experiences. The articles illustrate how these experiences complicate the practice of ethical principles such as ‘respect for persons’ within healthcare settings. While much work still needs to be done, we hope that these contributions will help to draw attention to the need for interdisciplinary engagement, for further understanding of these concepts and phenomena to advance both academic and healthcare practice debates.

## CONFLICT OF INTEREST

NA.

## Data Availability

NA.

## References

[1] Loughlin M , Upshur R , Goldenberg M , Bluhm R , Borgerson K . Philosophy, ethics, medicine and health care: the urgent need for critical practice. J Eval Clin Pract. 2010;16(2):249‐259. 20367844 10.1111/j.1365-2753.2010.01411.x

[2] Loughlin M , Bluhm R , Buetow S , et al. Virtue, progress and practice. J Eval Clin Pract. 2011;17(5):839‐846. 21951924 10.1111/j.1365-2753.2011.01748.x

[3] Loughlin M , Bluhm R , Buetow S , et al. Reason and value: making reasoning fit for practice. J Eval Clin Pract. 2012;18(5):929‐939. 22994987 10.1111/j.1365-2753.2012.01896.x

[4] Loughin M , Bluhm R , Buetow S , Borgerson K , Fuller J . Reasoning, evidence and clinical decision‐making: the great debate moves forward. J Eval Clin Pract. 2017;23(5):905‐914. 28960730 10.1111/jep.12831

[5] Loughlin M , Bluhm R , Stoyanov D , et al. Explanation, understanding, objectivity and experience. J Eval Clin Pract. 2013;19(3):415‐421. 23692221 10.1111/jep.12060

[6] Loughlin M , Bluhm R , Fuller J , et al. Philosophy, medicine and health care—where we have come from and where we are going. J Eval Clin Pract. 2014;20(6):902‐907. 25644615 10.1111/jep.12275

[7] Loughlin M , Mercuri M , Parvan A , Copeland S , Tonelli M , Buetow S . Treating real people: science and humanity. J Eval Clin Pract. 2018;24(5):919‐929. 30159956 10.1111/jep.13024

[8] Loughlin M , Buetow S , Cournoyea M , Copeland S , Chin‐Yee B , Fulford KWM . Interactions between persons—knowledge, decision‐making and the coproduction of practice. J Eval Clin Pract. 2019;25(6):911‐920. 31733025 10.1111/jep.13297

[9] Loughlin M . Decision‐making, reasoning, context and perspective. J Eval Clin Pract. 2020;26(1):387‐388. 32131147 10.1111/jep.13381

[10] Loughlin M , Bluhm R , Fuller J , et al. Diseases, patients and the epistemology of practice: mapping the borders of health, medicine and care. J Eval Clin Pract. 2015;21(3):357‐364. 25923823 10.1111/jep.12370

[11] Loughlin M , Copeland S . Humans, machines and decisions: clinical reasoning in the age of artificial intelligence, evidence‐based medicine and Covid‐19. J Eval Clin Pract. 2021;27(3):475‐477. 33890703 10.1111/jep.13572 PMC8251491

[12] Andersen M , Varga S , Folker A . On the definition of stigma. J Eval Clin Pract. 2022;28(5):847‐853. 10.1111/jep.13684 PMC9790447 35462457

[13] Dolezal L . Shame anxiety, stigma and clinical encounters. J Eval Clin Pract. 2022;28(5):854‐860. 10.1111/jep.13744 PMC7613638 35903848

[14] Hutchinson P . Stigma respecified: investigating HIV stigma as an interactional phenomenon. J Eval Clin Pract. 2022;28(5):861‐866. 10.1111/jep.13724 PMC9796448 35709243

[15] Fieller D , Loughlin M . Stigma, epistemic injustice and ‘looked after children’: the need for a new language. J Eval Clin Pract. 2022;28(5):867‐874. 10.1111/jep.13700 PMC9790323 35599388

[16] Heney D . Solving for stigma in mental health care. J Eval Clin Pract. 2022;28(5):883‐889. 10.1111/jep.13735 35809228

[17] Ballesteros V . A stigmatizing dilemma in the labor room: irrationality or selfishness? J Eval Clin Pract. 2022;28(5):875‐882. 10.1111/jep.13747 PMC9796655 35913362

[18] Smith RJ , Atkinson P , Evans R . Situating stigma: the case of accounting for deviancy, difference and categorical relations. J Eval Clin Pract. 2022;28(5):890‐896. 10.1111/jep.13749 PMC9804660 36006683

[19] Athanasios A , Lee K . Using analogy to facilitate patient education with borderline personality disorder: the borderline car. J Eval Clin Pract. 2022;28(5):897‐898. 10.1111/jep.13751 35945189

[20] Bromley E . Shame as a moral mood in medicine. J Eval Clin Pract. 2022;28(5):899‐908. 10.1111/jep.13708 PMC9796295 35655432

[21] Cheston K . (Dis)respect and shame in the context of ‘medically unexplained’ illness. J Eval Clin Pract. 2022;28(5):909‐916. 10.1111/jep.13740 PMC9796720 35899324

[22] Smith‐Oka V . You're joking: exploring humor and humiliation as forms of shame and obstetric violence within medical encounter. J Eval Clin Pract. 2022;28(5):917‐923. 10.1111/jep.13741 35871442

[23] Park S . A reflection on health and disease amid COVID‐19 pandemic. J Eval Clin Pract. 2022;28(5):711‐716. 10.1111/jep.13673 PMC9114899 35262996

[24] Pinto E , Turchi GP , Orrù L , Iudici A . A contribution towards health. J Eval Clin Pract. 2022;28(5):717‐720. 10.1111/jep.13732 PMC9796475 35771620

[25] Moe F , Berg H . Treating equivalent cases differently: a comparative analysis of substance use disorder and type 2 diabetes treatment guidelines. J Eval Clin Pract. 2022;28(5):721‐728. 10.1111/jep.13693 PMC9790317 35484825

[26] Krasin E . Karl Popper 120th anniversary: some reflections on the decision‐making process in medicine. J Eval Clin Pract. 2022;28(5):729‐730. 10.1111/jep.13603 34296496

[27] Garg PK , Pandey D . Do we need evidence for evidence‐based medicine? J Eval Clin Pract. 2022;28(5):731‐732. 10.1111/jep.13577 33963662

[28] Langlois‐Thérien T , Dewar B , Upshur R , Shamy M . Use of evidence in acute stroke decision‐making: implications for evidence‐based medicine. J Eval Clin Pract. 2022;28(5):733‐740. 10.1111/jep.13597 34258832

[29] Berg H , Askheim C , Heggen KM , Engebretsen E . From evidence‐based to sustainable healthcare: Cochrane revisited. J Eval Clin Pract. 2022;28(5):741‐744. 10.1111/jep.13698 PMC9790628 35570321

[30] Levack‐Payne W . Mechanistic evidence and exercise interventions: causal claims, extrapolation, and implementation. J Eval Clin Pract. 2022;28(5):745‐751. 10.1111/jep.13748 PMC9804705 35971196

[31] Landes J , de Pretis F , Jukola S . E‐synthesis for carcinogenicity assessments: a case study of processed meat. J Eval Clin Pract. 2022;28(5):752‐772. 10.1111/jep.13697 35754297

[32] Pel E , Engelberts I , Schermer MHN . Diversity of interpretations of the concept ‘patient‐centered care for breast cancer patients’; a scoping review of current literature. J Eval Clin Pract. 2022;28(5):773‐793. 10.1111/jep.13584 PMC9788211 34002460

[33] Lefkowitz A , Vizza J , Kuper A . Patients as experts in the illness experience: implications for the ethics of patient involvement in health professions education. J Eval Clin Pract. 2022;28(5):794‐800. 10.1111/jep.13672 35274414

[34] Telles‐Correia D . Values in mental and medical disorder concepts: their presence is not the point, being aware of them is. J Eval Clin Pract. 2022;28(5):801‐806. 10.1111/jep.13679 35445481

[35] Radden J . Capturing the anorexia nervosa phenotype: conceptual and normative issues in ICD‐11. J Eval Clin Pract. 2022;28(5):807‐813. 10.1111/jep.13586 34121277

[36] Elton L . Epistemic risk and non‐epistemic values in end of life care. J Eval Clin Pract. 2022;28(5):814‐820. 10.1111/jep.13675 35286006

[37] Hubbeling D . Overtreatment: is a solution possible? J Eval Clin Pract. 2022;28(5):821‐827. 10.1111/jep.13632 PMC9786860 34693594

[38] Wahl A , Andersen M , Ødemark J , Reisæther A , Urstad K , Engebretsen E . The importance of shared meaning making for sustainable knowledge translation and health literacy. An example from kidney transplantation. J Eval Clin Pract. 2022;28(5):828‐834. 10.1111/jep.13690 PMC9790374 35466469

[39] Pietarinen A‐V , Stanley D . Precision medicine and diseases as natural kinds: an epistemological dilemma. J Eval Clin Pract. 2022;28(5):835‐842. 10.1111/jep.13707 35644924

[40] Buetow S , Zawaly K . Rethinking researcher bias in health research. J Eval Clin Pract. 2022;28(5):843‐846. 10.1111/jep.13622 34590758

[41] Goffman E . Stigma: Notes on the Management of Spoiled Identity. Prentice‐Hall; 1963.

[42] Clair M . Stigma. In: Ryan JM , ed. Core Concepts in Sociology. Wiley‐Blackwell; 2018.

[43] Hebl MR , Dovidio JF . Promoting the “social” in the examination of social stigmas. Pers Soc Psychol Rev. 2005;9(2):156‐182. 10.1207/s15327957pspr0902_4 15869380

[44] Parker R , Aggleton P . HIV and AIDS‐related stigma and discrimination: a conceptual framework and implications for action. Soc Sci Med. 2003;57(1):13‐24. 10.1016/S0277-9536(02)00304-0 12753813

[45] Scambler GA . Sociology of Shame and Blame: Insiders Versus Outsiders. Palgrave Macmillan; 2020.

[46] Tyler I , Slater T . Rethinking the sociology of stigma. Sociol Rev. 2018;66(4):721‐743. 10.1177/0038026118777425

[47] Hutchinson P , Dhairyawan R . Shame and HIV: strategies for addressing the negative impact shame has on public health and diagnosis and treatment of HIV. Bioethics. 2017;32(1):68‐76. 10.1111/bioe.12378 28833363 PMC5763398

[48] Hutchinson P , Dhairyawan R . Shame, stigma, HIV: philosophical reflections. Med Humanit. 2017;43(4):225‐230. 10.1136/medhum-2016-011179 28790137 PMC5739830

[49] Barrett LF . Solving the emotion paradox: categorization and the experience of emotion. Person Soc Psychol Rev. 2006;10(1):20‐46. 10.1207/s15327957pspr1001_2 16430327

[50] Barrett LF . How Emotions Are Made: The Secret Life of the Brain. Macmillan; 2017.

[51] Leys R . The Ascent of Affect: Genealogy and Critique. University of Chicago Press; 2018.

[52] Taylor G . Pride, Shame, and Guilt: Emotions of Self‐Assessment. Clarendon Press; 1985.

[53] Nussbaum M . Emotions as judgments of value and importance. In: Solomon RC , ed. Thinking About Feeling: Contemporary Philosophers on Emotions. Oxford University Press; 2004:183‐199.

[54] Solomon RC . Not Passion's Slave: Emotions and Choice. Oxford University Press; 2006.

[55] Roberts RC . Emotions: An Essay in Aid of Moral Psychology. Cambridge University Press; 2003.

[56] Deigh J . Cognitivism in the theory of emotions. Ethics. 1994;104(4):824‐854. 10.1086/293657

[57] Griffiths PE . What Emotions Really Are: The Problem of Psychological Categories. University of Chicago Press; 1997. 10.7208/chicago/9780226308760.001.0001

[58] Hutchinson P . Shame and Philosophy: An Investigation in the Philosophy of Emotions and Ethics. Palgrave Macmillan; 2008. 10.1007/s11158-010-9120-4

[59] Hutchinson P . The “placebo” paradox and the emotion paradox: challenges to psychological explanation. Theory Psychol. 2020;30:617‐637. 10.1177/0959354320928139

[60] Dolezal L . The Body and Shame: Phenomenology, Feminism, and the Socially Shaped Body. Vol 4. Lexington Books; 2015.

[61] Colombetti G . The Feeling Body: Affective Science Meets the Enactive Mind. MIT Press; 2014. 10.7551/mitpress/9780262019958.001.0001

[62] Dillon RS . Respect. In: Zalta EN , ed. *The Stanford Encyclopedia of Philosophy*. Fall 2022. Metaphysics Research Lab, Stanford University; 2022.

[63] Dillon RS . Respect: a philosophical perspective. Gruppendynamik. 2007;38(2):201‐212. 10.1007/s11612-007-0016-5

[64] Subramani S , Biller‐Andorno N . Revisiting respect for persons: conceptual analysis and implications for clinical practice. Med Health Care Philos. Published online April 4, 2022. 10.1007/s11019-022-10079-y PMC8994924 35397708

[65] Lysaught MT . Respect: or, how respect for persons became respect for autonomy. J Med Philos. 2004;29(6):665‐680. 10.1080/03605310490883028 15590515

[66] Darwall SL . Two kinds of respect. Ethics. 1977;88(1):36‐49. 10.1086/292054

[67] Cavell S . The Claim of Reason. Oxford University Press; 1979.

[68] Millum J , Bromwich D . Respect for persons. In: Iltis AS , MacKay D , eds. The Oxford Handbook of Research Ethics. Oxford University Press; 2020:1‐30.

[69] Beach MC , Duggan PS , Cassel CK , Geller G . What does ‘respect’ mean? exploring the moral obligation of health professionals to respect patients. J Gen Intern Med. 2007;22(5):692‐695. 10.1007/s11606-006-0054-7 17443381 PMC1852905

[70] Brännmark J . Respect for persons in bioethics: towards a human rights‐based account. Hum Rights Rev. 2017;18(2):171‐187. 10.1007/s12142-017-0450-x

[71] Dolezal L , Lyons B . Health‐related shame: an affective determinant of health? Med Humanit. 2017;43(4):257‐263. 10.1136/medhum-2017-011186 28596218 PMC5739839

